# TalkingMats as a decision aid to promote involvement in choice and decision-making around home care services for older people with mild to moderate dementia – study protocol for a randomized controlled trial

**DOI:** 10.1186/s12877-023-03956-5

**Published:** 2023-04-21

**Authors:** Anna Dunér, Angela Bångsbo, Tina M. Olsson

**Affiliations:** 1 Department of Social Work, University of Gothenburg, PO Box 720, SE 405 30 Göteborg, Sweden; 2 Department of Work Life and Social Welfare, University of Borås, Borås, Sweden; 3 School of Health and Welfare, Jönköping University, Jönköping, Sweden

**Keywords:** Decision aid, Dementia, Home care services, Involvement, Randomized controlled trial

## Abstract

**Background:**

In Sweden, 72% of people with dementia live in ordinary housing. Of these, 50% receive home care services. Older people with dementia may benefit from developments in decision-making support which aim to facilitate their ability to communicate their personal needs and preferences with care managers and staff in home care services. In this study, we will test and evaluate the use of TalkingMats in Swedish municipal home care services for older people with mild to moderate dementia. TalkingMats is a low-technology communication tool, to help people with communication difficulties express their views. It uses a simple system of picture symbols which are placed on a textured mat. This study will provide insight into the extent to which TalkingMats benefits older people with dementia to feel more involved in decisions related to home care services. In addition, this study will assess the extent to which the use of TalkingMats promotes service providers’ efforts to involve service recipients in decision making. The implementation of TalkingMats in home care services will also be studied.

**Methods:**

A parallel group, two-armed randomized controlled trial design in which TalkingMats and Usual Conversation Method will be compared. Two specific situations where older people with dementia must make decisions about home care services will be studied. First, a follow-up needs-assessment conversation between study participants and care managers will be studied. Second, a conversation between participants and home care staff regarding the delivery of the decided home care services will be studied. In addition, a qualitative approach will be used to gain an understanding of study participant and service provider experiences of the impact and implementation of TalkingMats.

**Discussion:**

The combined exploratory, descriptive, and experimental study design is considered an important strength which will facilitate multi-facetted knowledge production concerning the involvement and communication needs of older people with dementia generally and within the context of home care services specifically. Combining qualitative and quantitative methods will maximize our ability to assess the effects of TalkingMats.

**Trial registration:**

ClinicalTrials.gov ID: NCT05561998. Registered in September 28, 2022.

## Background

In this study we will test and evaluate the use of TalkingMats (TM) as a decision aid in conversations between home care service providers and older people with mild to moderate dementia within Swedish municipal eldercare. In Sweden, approximately 160,000 people live with dementia. In addition, it is estimated that half of the population over 90 years of age live with dementia [1]. In a Swedish study, Odzakovic et al. [2] found that of those with dementia, 72% were living in ordinary housing. Of these, 50% were receiving home care services. In Sweden, when an older person is no longer able to manage daily independent living, they can apply for assistance from the municipal home care services. The extent of the support ultimately provided through home care services is based on a needs assessment. Needs assessments are performed by municipal care managers. If eligible, home care services may include for example help with household chores, personal care, and/or social activities. The purpose of the present study is to increase our understanding of how TM impacts the involvement of older people with dementia in decisions made within the context of home care services.

During the last decade, developments in Swedish eldercare have been dominated by a drive for individualized support with an emphasis on consumer choice [3, 4]. The intent of increased consumer choice being not only to increase choice and control for individuals receiving services, but also to increase the ability to customize services for individual service recipients and increase the quality of the services provided. The Swedish Social Services Act [5] states that eldercare should aim at strengthening older people’s ability to live an independent life, in dignity, and with well-being [4]. Additionally, the Swedish National guidelines on dementia care [1] stipulate that people with dementia are covered by the same policy intentions as other groups using eldercare.

Needs-assessments and conversations about the provision of home care services for older people with impaired cognition are especially challenging [6]. Care managers who conduct needs-assessments and make decisions about home care services often lack training in communicating with people with dementia or other cognitive impairments. In addition, service recipients often lack access to information regarding the services available to them [7]. Home care staff who deliver support often express difficulties with interpreting the wishes of older people with impaired cognition or dementia [8]. In contrast to other countries, Sweden lacks decision-making support for older people making choices about social care [9, 10]. The lack of opportunities for supported decision-making may force service users with the most complex needs into passivity. However, such support may facilitate the ability of older people with dementia to communicate their needs and preferences with home care service providers more clearly [6–8].

Research on people with dementia, using a social perspective focusing on citizenship and rights, has shown that people with dementia, even in advanced stages, can express their wishes and preferences in alternative, more creative ways [11, 12]. In addition, care managers’ behavior in professional encounters with older people with dementia can either promote or constrain self-determination [13]. Studies have found that care managers face several dilemmas in the assessment of the need for support for people with dementia [7, 14]. In addition, families can confuse the best interests of service users with their own interests, which raises questions regarding undue influence [15–17]. Rather than being involved, people with dementia are frequently excluded from decisions about their future care and are often talked about rather than talked to [18, 19]. In such situations, methods to support decision-making may be required. This would allow service users with dementia increased opportunity for choice and control [10, 20, 21] in decisions regarding themselves. A recent meta-analysis of decision aids for older adults showed that decision aids increased knowledge, created more accurate risk perceptions, and helped participants choose options more congruent with their own values [22]. Many of these decision aids were designed as visual aids to be used as conversation props. Even though previous research has shown that such decision support would be welcomed by care managers and other staff in eldercare [7, 8], the use of decision aids when communicating with older people with dementia is extremely limited in Swedish eldercare. This points to the importance of developing and evaluating communication tools and decision aids for use in conversations regarding needs-assessment and of the delivery of home care services.

In this study, we will test TM, an established communication tool, as a decision aid in conversations regarding needs-assessments and the delivery of home care services. In previous research, TM has shown promising results in its ability to support communication with people with dementia. In studies involving people with dementia, TM has been found to positively impact participants perception of involvement in decision-making and choice about things that matter to them [11, 23, 24]. Additionally, TM has been shown to improve participants with dementia's ability to understand conversation topics by providing a visual cue which allows more time to process information [24]. Thus, TM may play an important role in improving quality of care by providing a tool that home care service providers can use to engage with people with dementia and help them express their views about a range of topics [23].

### Aims and research questions

The aim of this study is to test and evaluate the use of TM as a decision aid in conversations regarding needs assessment and the delivery of home care services for older people with mild to moderate dementia within Swedish municipal eldercare. Specifically, this study will provide more insight into the extent to which TM impacts participants perception of their involvement in decision making and the extent to which TM promotes service providers’ efforts to involve service recipients in decision making. Moreover, the process by which TM is implemented into normal municipal eldercare will be studied closely. Using a mix of quantitative and a qualitative designs the following research questions will be answered:

- RQ1. To what extent does TM impact participants’ perceived involvement in decision making in conversations regarding home care services compared with usual conversation methods (UCM)?
- RQ2. To what extent does TM impact service provider efforts to involve participants in decision making in conversations regarding home care services compared with UCM?
- RQ3. What are the barriers and facilitators of implementing TM as a decision aid in the context of Swedish municipal home care services for older people with mild to moderate dementia?

## Methods

### Design and setting

**Fig. 1:**
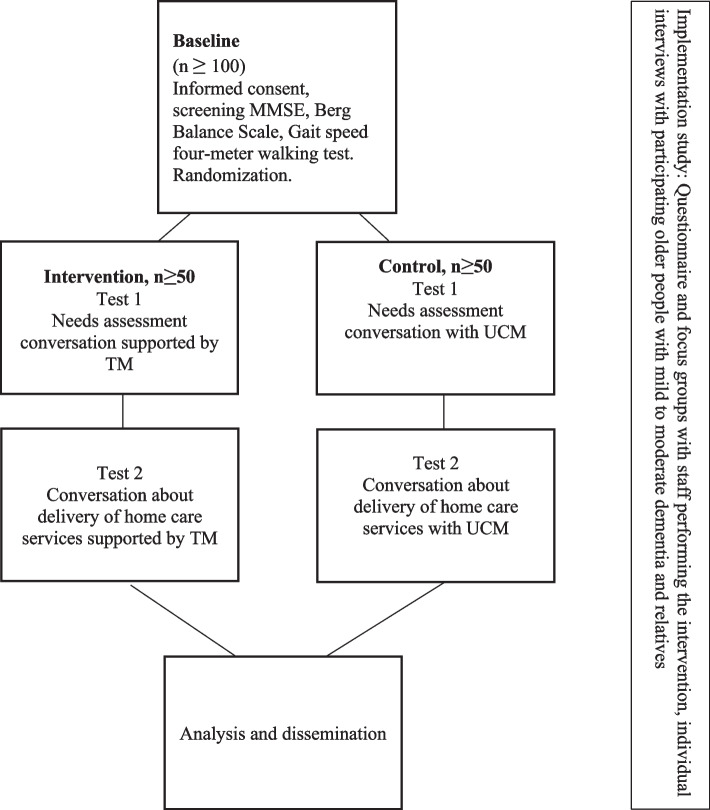
Overview over the design of the study

The present study (Fig. 1) uses exploratory, descriptive, and experimental methods*.* The study will be conducted within the normal operating Swedish municipal eldercare services. The parallel group intervention study is designed as a two-armed randomized controlled trial (RCT) where the use of TM as a decision aid to support involvement in conversations regarding home care services for people with mild to moderate dementia will be evaluated through comparison with UCM. Two different situations where older people with dementia must make decisions about home care services will be studied. The first situation is a needs-assessment conversation between care managers working in eldercare and participants which concerns the follow-up of decisions about home care services. The second situation is a conversation between home care staff and participants regarding the delivery of the home care services chosen during the needs assessment. This study builds upon a previously conducted pilot study (*N* = 10) which aimed to test the intervention, study inclusion criteria, research and practice logistics, and outcome measurements prior to the full effectiveness study described here.

This study will take place within the local eldercare authorities of three municipalities in southwestern Sweden. The largest municipality has just over 100,000 inhabitants and about 22,000 inhabitants aged 65 + . The two smaller municipalities have just under 47 000 inhabitants combined. Approximately 11,000 of whom are aged 65 + . The study will be performed in collaboration between researchers from the Department of Social Work at the University of Gothenburg, the Department of Work Life and Social Welfare at the University of Borås, the School of Health and Welfare at Jönköping University, and managers, research assistants and staff from three municipal eldercare authorities. Continuous meetings and workshops with collaborators will be held throughout the study period, to monitor participant enrolment and adherence to study protocols.

### Recruitment and eligibility criteria

The target group for this study is older people with mild to moderate dementia who use home care services provided by municipal eldercare authorities. Service users will be identified by care managers, healthcare staff or a specialized dementia team. At first contact, potential participants will receive brief information about the study and be offered the opportunity to participate in screening tests for mild to moderate dementia. It will be made clear that the choice to participate in the study (or not) will not affect receipt of eldercare. On expression of interest to participate in the study, permission will be sought by home care service providers to forward contact details to the research team. A research assistant will contact individuals referred to the study to arrange a convenient time to meet. At this visit, written and verbal informed consent will be obtained, and baseline data will be collected.

Eligibility criteria:

- Participants are aged 65 or older.
- Participants receive home care services from the eldercare authority in one of the three participating municipalities.
- Participants are assessed as having mild to moderate dementia which is defined as scoring between 12–23 on the mini-mental state examination (MMSE).

According to preliminary power calculations, we plan to include 50 participants in each study arm. The calculation was made based on what is needed to detect a moderate difference (0.25 effect size) with 0.9 power, p = 0.05 [25]. Significant differences over time in involvement were found using the Involvement Measure Scale (IMS) in a sample of 18 in a previous study [11].

### Time plan

The inclusion of participants started in November 2022. Interviews and focus groups will be conducted between January 2023 and December 2024. Inclusion as well as data collection is estimated to be completed in December 2024.

### Allocation and blinding

Eligible participants will be randomized to either the intervention (TM) or control (UCM) group. The randomization schedule will be developed by a member of the research team not involved in participant recruitment or contact with referring agencies. The randomization sequence will be developed a priori by site (*n* = 3) using the online randomizer random.org with 50% allocation between arms. Results of the randomization for individual participants will be communicated following inclusion and collection of baseline measurement data.

Participants, service providers, and research staff will not be blinded to final participant allocation. Data collection subsequent to baseline measurements will not be blinded.

### Participant timeline

**Fig. 2:**
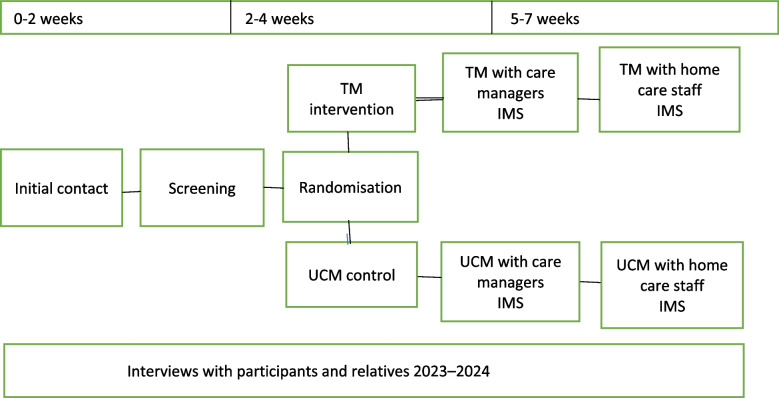
Schedule of enrolment, interventions, and assessments

Study participants will be offered the screening test within two weeks from their first contact with research staff (Fig. 2). Following screening, study participants will be randomized to TM or UCM. Two weeks following screening, participants will have a needs assessment conversation with a care manager. Approximately three weeks post conversation with care managers, a meeting with home care service staff will take place. Directly following participants' conversation with care managers and home care staff, a researcher will conduct a standardized interview using the IMS. In 2023 or 2024, participants as well as their relatives will be invited to take part in qualitative interviews with one of the researchers.

### Interventions

#### TalkingMats (TM) intervention

Participants in the intervention group will use TM as a decision aid in needs assessment conversations as well as in conversations about the delivery of home care services. TM is a low-technology communication framework, designed to help people with communication difficulties express their views and was developed through research conducted at the University of Stirling in Scotland. TM uses a simple system of picture symbols which are placed on a textured mat. TM allows people to indicate their feelings about various options within a topic by placing the relevant image below a visual scale. Three sets of symbols are used. One set of symbols is used for the topics of the conversation, one set for the options within each topic, and one set consisting of a value scale under which the options are placed. Previous studies [11, 26] have found that the TM could be used by many people at all stages of dementia and that the use of TM improved participants’ ability to communicate compared with usual conversation methods. The use of TM allowed participants with dementia to communicate their needs and preferences with greater ease, helped them remain in control of their own daily living activities, and assisted them when facing difficult decisions.

In a previous pilot study conducted by our research team, all TM materials and procedures were adapted in collaboration with service providers in one of the municipalities participating in the present study. In addition, specific TM material was developed and adapted for the purpose of the present study. The specific topics and range of options within each topic for use within the TM framework have been identified in workshops with service providers using the World Health Organization’s International Classification of Functioning (ICF; [27]) and in line with the systematic needs-assessment tool “The Individual’s Needs in the Center” (IBIC) [28]. The material and procedures to be used in the present study have been tested, revised, and re-tested several times in preparation for this study. In September 2020, researchers and service providers tested the TM with older adults (acquaintances and/or service users). Subsequently, care managers, home care staff, and researchers tested and revised the TM material in reoccurring workshops held between autumn 2020 through 2021. All instructions and TM materials have been gathered in binders which have been provided to all care managers, home care staff, and research assistants involved in the study. All service providers and research assistants involved in the pilot study as well as the present study have received a two-day training in the use of TM from accredited TM trainers at the Dart Communication and Data Resource Centre, Region Västra Götaland, Sweden.

#### Control group

Participants in the control group will receive UCM for both the needs-assessment conversation and the conversations about home care service provision. The needs-assessment conversations are based on the systematic needs-assessment tool IBIC [28].

### Outcomes

#### Primary outcome measure—participants

##### Involvement Measure Scale (IMS)

The IMS measures older people’s involvement in conversations around care. IMS is made up of six questions from the Freedom of Choice Interview Schedule [29] which is a measure specifically designed for people with dementia and their family carers to indicate how they rate their involvement in a situation where they had to consider issues around care. Participants rate questions on a 4-point scale ranging from “never/none” to “always/all”. When evaluating the use of TM for people with dementia, Murphy et al. [11] used a slightly adapted IMS, that we have translated to Swedish for the purpose of the present study [30].

#### Primary outcome measure – service providers

##### OPTION scale

Service provider efforts to involve older people with mild to moderate dementia in conversations around needs-assessments and service delivery are measured with the OPTION- scale which has been found valid and reliable in previous research [31]. The scale is made up of 12 questions and has been translated for the present study. The 12-items are rated on a five-point scale ranging from participant “is not involved” to participant “is involved to a high degree”.

#### Implementation measures

Implementation will be studied in conjunction with the effectiveness study. Clinical encounters will be evaluated through observations of video recordings, using a fidelity checklist developed in the pilot study. In addition, a survey will be administered to the staff performing the intervention. Finally, exploratory qualitative interviews will be performed with service providers and study participants to gain an understanding of their perception of TM as well as of the quality of the implementation of TM.

The implementation survey consists of three implementation outcome measures found valid and reliable in previous research [32]:

##### Acceptability of Intervention Measure (AIM)

The AIM is a four-item scale that measures the perception among implementation stakeholders that a given treatment, service, practice, or innovation is agreeable, palatable, or satisfactory. Items are rated on a 5-point scale from “completely disagree” to “completely agree”.

##### Intervention Appropriateness Measure (IAM)

The IAM is a four-item scale that measures the perceived fit, relevance, or compatibility of the innovation or evidence-based practice for a given practice setting, provider, or consumer, and/or perceived fit of the innovation to address a particular issue or problem. Items are rated on a five-point scale from “completely disagree” to “completely agree”.

##### Feasibility of Intervention Measure (FIM)

The FIM is a four-item scale that measures the extent to which a new treatment, or an innovation, can be successfully used or carried out within a given agency or setting. Items are rated on a five-point scale from “completely disagree” to “completely agree”.

### Data collection methods

#### Baseline data

Baseline data includes background variables such as age, gender, educational background, living situation, and the cognitive and functional ability of participants. Cognitive and functional ability will be measured using the MMSE [33], the Berg Balance Scale [34], and the Gait speed four-meter walking test [35]. This data will be collected by the study´s research assistants at an initial visit to the study participants home. The research assistants in the study are occupational therapists, registered nurses, or physiotherapists familiar with the process of obtaining informed consent as well as of using the baseline measurement instruments and tests.

#### Outcome data

Primary outcome data will be collected by the research team. Data on participant involvement in conversations around care, measured with the IMS, will be collected at two time points (T1, T2). T1 will be conducted directly after the needs assessment conversation and T2 will be conducted directly after the conversations about delivery of home care services. Data on service providers' efforts to involve participants in conversation will be obtained through video-recordings of the conversations. These recordings will be evaluated by the researchers using the OPTION scale.

The secondary outcome data will be collected through a survey to care managers and staff performing the intervention, consisting of the three implementation outcome measures AIM, IAM, and FIM. The survey will be administered at baseline (directly after the TM training), after 6 months, and after 12 months. Data on implementation fidelity will be obtained by evaluations of the video recorded clinical encounters, using the above-mentioned checklist.

#### Qualitative data

A qualitative approach will be used to gain an understanding of study participant and service provider experiences of using TM as well as their perceptions regarding its impact and implementation. Both individual interviews and focus groups will be conducted. Exploratory qualitative interviews will be performed with study participants individually or, when appropriate, with their relatives. Focus groups will be performed with service providers using TM. Focus group discussions and individual interviews will be audio taped and transcribed verbatim.

### Data management

Data will be processed so that unauthorized persons cannot access it. The material will be pseudonymized and stored using the University of Gothenburg's service for secure data storage for class 3 data. Security functions for class 3 data storage include two-factor authentication. Results from screening tests and outcome measures will be documented on special forms for each participant. The coded results are then entered into IBM SPSS Statistics for Windows version, 28.0 (IBM Corp., Armond, New York). The code key will be stored separately from other material. The video recordings will be transferred to secure data storage and the original files will be destroyed. When the project is finished, the collected material will be saved for a period of 10 years to enable review of the data in accordance with the University of Gothenburg's rules for handling and archiving research documents. In accordance with the Swedish Archives Act [36], the material will be archived and accessible and if the research material is not judged to have value in terms of the criteria set forth in the National Archives' regulation it will be deleted in 10 years.

### Analysis

Study data will be pooled with pilot data for analysis. To answer RQ1 and RQ2, we will perform descriptive and comparative statistical analyses as well as qualitative thematic analysis [37]. Statistical data analysis methods will be chosen based on data characteristics (e.g., ANOVA, Kruskal–Wallis). Baseline differences between groups will be controlled for in the final analyses. Sensitivity analyses will be conducted (e.g., TOT vs ITT analysis). Methods for handling missing data will be determined based on a missing data analysis to determine the nature of the missingness (e.g., MCAR). Fidelity and implementation will be tested for their impact on results. To check for comparison group equivalence, we will compare the pre-test dementia scores, functional ability and demographic variables of the two groups. When evaluating the video-recordings of the conversations, using the “OPTION scale”, inter-rater reliability will be tested. To interpret the effects of the intervention, qualitative thematic analyses [37] of the participants’ and staff’s experiences as expressed in focus group and individual interview data will be conducted.

For RQ3 we will perform descriptive analyses of survey-data as well as qualitative thematic analysis of focus group and individual interview data. This will provide analysis of patterns and themes in the interview and focus group statements. The qualitative data will enable an analysis of similarities as well as differences in the experiences of the persons involved in the study, on an individual as well as a group level. In addition, a mixed method approach [38] will be used linking descriptive statistics and qualitative analysis.

### Dissemination

Results from the study will be communicated within the research community and with home care service practitioners, policy makers, and user representatives. The research community will be reached through participation in international research conferences and publication in peer-reviewed scientific journals. In addition, results will be presented to and discussed with practitioners, policy makers, and service users via conferences arranged by local Research & Development units, their national association in the area of social welfare, and the National Dementia Association. We will also communicate study results through open access publications.

## Discussion

The study “Talking Mats as Decision Aid to Promote Involvement in Choice and Decision-Making around Home Care Services for Older People with Mild to Moderate Dementia” evaluates the extent to which the TM decision aid is more effective than UCM in supporting the involvement of older people with mild to moderate dementia in decisions and choices around home care services. As people with dementia are frequently excluded from decisions about their future care and are often talked about, rather than talked to [18, 19], the opportunity for increased involvement in such discussions is imperative for the creation of equal opportunity for this group.

The combined exploratory and experimental study design is considered an important strength and will facilitate multi-facetted knowledge production. Combining qualitative and quantitative methods could maximize the ability to bring different perspectives together in a research project and give unique opportunities to better understand the processes involved in improving participation of older adults with mild to moderate dementia in decision making around home care services. Such knowledge will have the capacity to advance both the science of communication and improve the practice of communicating with older persons with dementia. Moreover, this study has the potential to impact how we educate students in professions that meet older people with dementia.

To enhance the quality of the present study, we have conducted a pilot study with the purpose of pre-testing the TM communication tool, study inclusion criteria, and study logistics. Managers and staff from eldercare in one local authority participated in the development of the TM inclusion criteria, and study logistics through continuous meetings with researchers in the joint project steering group. Furthermore, workshops with care managers and home care staff were held to discuss and develop the TM material for these specific purposes. The idea was to adopt a pragmatic approach, balancing sound research methodology with clinical relevance and usefulness. Nevertheless, it is important to distinguish between those involved in the intervention and those conducting research on the intervention. In this study, service providers will be responsible for the implementation and delivery of TM and the research team will be responsible for data collection and analysis. Due to the Covid-19 pandemic, we were not able to include the perspective of “experts by experience” i.e., older people with dementia and their relatives in the planning of the study. However, in the present study, the research team will involve municipal dementia teams to recruit interested reference people from the target population who are willing to provide continuous input during the performance of the study.

The nature of the study involves several ethical concerns. Participants with mild to moderate dementia are in different situations of dependency and vulnerability. Still, we find it important that this group is given the same opportunity to participate in research studies as is given other populations, if they are willing and able to do so. This requires ethical awareness and preparedness from the research team to continuously assess possible ethical concerns. Our research team has vast previous experience dealing with ethical issues regarding research in relation to potentially vulnerable and dependent groups, including older adults with mild to moderate dementia and populations receiving home care services.

## Data Availability

Some of the datasets generated and analyzed during the current study will be available from the corresponding author upon reasonable request. However, video recordings of clinical encounters and some of the qualitative data will not be publicly shared due to the nature of the information which could compromise research participant privacy.
